# Biodiversity and Spatiotemporal Variation of Longhorn Beetles (Coleoptera: Cerambycidae) in Tropical Forest of Thailand

**DOI:** 10.3390/insects12010045

**Published:** 2021-01-08

**Authors:** Sirapat Yotkham, Piyawan Suttiprapan, Natdanai Likhitrakarn, Chayanit Sulin, Wichai Srisuka

**Affiliations:** 1 Department of Entomology, Faculty of Agriculture, Chiang Mai University, Chiang Mai 50200, Thailand; Sirapaty27@gmail.com; 2 Innovative Agriculture Research Center, Faculty of Agriculture, Chiang Mai University, Chiang Mai 50200, Thailand; 3 Division of Plant Protection, Faculty of Agricultural Production, Maejo University, Chiang Mai 50290, Thailand; kongerrrr@hotmail.com; 4 Entomology Section, Queen Sirikit Botanic Garden, P.O. Box 7, Chiang Mai 50180, Thailand; chayanitsulin@gmail.com

**Keywords:** Cerambycidae, longhorn beetles, biodiversity, distribution, tropical rain forest

## Abstract

**Simple Summary:**

Longhorn beetles are a large family of beetles and have a wide-geographic distribution. Some of them are pests of many economic plants and invasive species. They also play roles in decomposition and nutrient cycling in forest ecosystems. They feed on living, dying, or dead woody plants in the larval stage. So far, 308 species of longhorn beetles have been reported from northern Thailand. However, the biodiversity and distribution of longhorn beetles in different elevation gradients and seasons, associated with environmental factors across six regions in the country, has not yet been investigated. In this study, longhorn beetle specimens were collected by malaise trap from 41 localities in 24 national parks across six regions in Thailand. A total of 199 morphospecies were identified from 1376 specimens. Seasonal species richness and abundance of longhorn beetles peaked during the hot and early rainy season in five regions, except for the southern region, which peaked in the rainy season. Our finding revealed that most species’ distribution was correlated with the region and forest type (at middle and low elevations). Quantitative data from this study can be useful to manage agricultural and forest plantations.

**Abstract:**

Longhorn beetles are highly diversified and important for agriculture and health of the environment. However, the fauna and ecology of these beetles are not well known in Thailand. This study is the first to report the biodiversity, elevation, and seasonal distribution of longhorn beetles. Specimens were collected by malaise traps from 41 localities in 24 national parks throughout the country during 2006–2009. The traps were operated at each site for 12 consecutive months with a monthly service. A total of 199 morphotaxa in 36 tribes of 6 subfamilies were identified from 1376 specimens. Of these, 40.7% and 14.5% of total taxa were singletons and doubletons, respectively. The Shannon diversity index and observed species richness at Panernthung, Loei Forest Unit and Mae Fang Hotspring were high at 0.96 (30), 0.88 (50), and 0.86 (34), respectively. Local richness ranged between 3 and 50 species, while the species richness estimator showed between 6 and 1275 species. The most relatively abundant species, *Nupserha lenita*, *Pterolophia* sp.1, *Oberea* sp.3, *Acalolepta pseudospeciosa,* and *Ac. rustricatrix* represented 4.80%, 4.80%, 4.80%, 4.5%, and 4.43% of the species, respectively. The species with the widest distribution range of percentage of species occurrence (% SO) was *Pt.* sp.1 (63.4%), followed by *Ac. rustricatrix* (39%) and *Moechotypa suffusa* (39%). In a significantly negative relationship between species richness and elevation (*p* > 0.05, R^2^ = 0.04), the species richness pattern showed a hump-shaped curve that peaked at the middle elevation (501–1000 m asl). Regarding seasonal variation, most of the species occurred during the hot season (March–April) and peaked in early rainy season (May), while a low number of species were found during the mid-rainy (June–October) and cold season (November–February). Ordination analysis indicated that the distribution of most species was associated with regions and forest type, and most of the species correlated with forest located at middle and low elevation. The results of this study indicated the very high biodiversity of longhorn beetles in Thailand, which suggests that an understanding of their seasonal and elevational distribution will be of value to agriculture management and conservation. They also indicated that malaise traps are appropriate for the evaluation of biodiversity.

## 1. Introduction

Understanding the biodiversity and ecology of insects, especially their seasonal activity and distribution patterns that are the largest components in the ecosystem, is necessary due to their impact on agriculture and economics. Beetles are largest order of insects, and longhorn beetles (Cerambycidae) are one of the larger families, with a wide-geographic distribution and more than 37,000 species in 5000 genera recorded. This group of beetles is charismatic, has high biodiversity with various ecological niches, and is of significant economic importance. Most of the members are phytophagous or xylophagous, with many species being pests of agricultural crops, ornamental trees and lumber products, thus causing millions of dollars in damage each year [1,2,3,4]. Approximately 200 species worldwide have been reported to have impact on agriculture, forestry, and horticulture, causing production losses by destroying plants by direct feeding or transmission of plant diseases [1]. Moreover, many longhorn beetles are invasive species with wide-range distribution [5]. Exotic invasive species have the potential to become devastating pests because they are difficult and costly to detect. A total of 19 exotic invasive longhorn beetle species have been reported in Europe, America, and Canada [6,7]. The Asian native species, *Psacothea hilaris hilaris* (Cerambycidae: Lamiinae), has been reported as an exotic invasive pest of *Morus,* mulberry and *Ficus* trees in Italy and Germany [8]. Some longhorn beetle species are reported as being agriculture and eco-friendly: *Alosterna tabacicolor* is the main pollinator of Orchid (*Dactylorhiza fuchsii*) in Poland [9], and during the larval stage, longhorn beetles play a critical role in nutrient cycling in forests [7]. In addition, morphological study and material composition of larvae mouthparts have been performed in some species, which could be applied for developing bionic technologies and structural concepts [10]. For example, secretion from the jaws of longhorn beetle larvae can be applied for developing wood preservatives [10]. However, longhorn beetles also cause economic loss in agriculture and forestry. Thus, a better understanding of their ecology, distribution, and environment relationship is necessary for preventing this problem. 

Although several studies have been carried out in many countries on the biodiversity and ecology of longhorn beetles in many aspects, such as host/plant relationship, distribution, seasonal activity of some economic importance species [11,12,13,14,15,16,17,18], the biodiversity of these beetles in Thailand is unknown. Over 10,000 insect species and 151 longhorn beetle species were catalogued from this country [19]. However, there have been no reports on the biodiversity or ecology of longhorn beetles in Thailand, and only three previous taxonomic studies: the first being in 1973 of 62 species reported in the northeast [20], the second in 1975 of 87 species discovered in Chiang Mai province, in the north [21], and the third in 2011 of 308 species also reported from the north, including 86 newly recorded species for the country [22]. Thus, this is the first report on the biodiversity and ecology of longhorn beetles in Thailand. The objective of this study was to analyze the biodiversity and distribution of longhorn beetles in spatial and temporal variation, associated with environmental factors across an elevation gradient and seasons in six regions of Thailand.

## 2. Materials and Methods

### 2.1. Study Area

**Figure 1:**
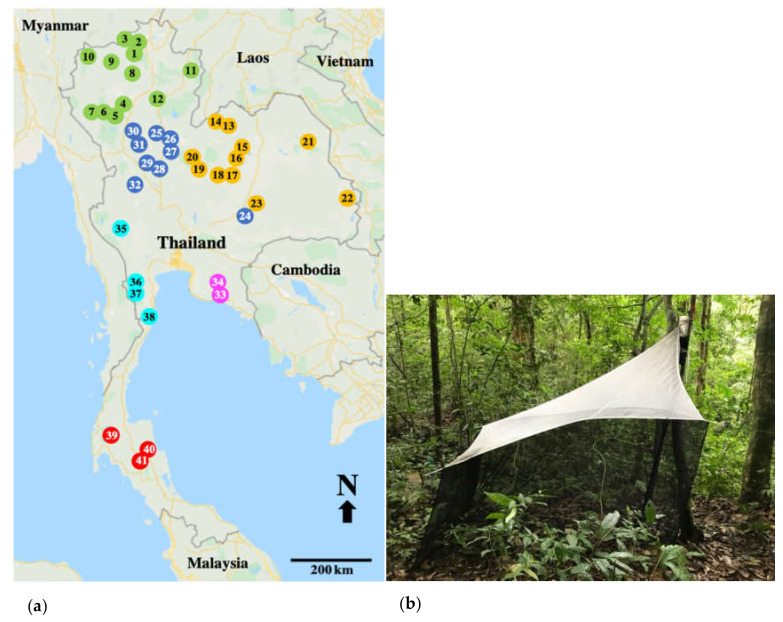
(**a**) Map of the study sites showing 41 collection sites for longhorn beetles in 6 regions of Thailand. Closed circles indicate the regions; green (1–12) = northern; orange (13–23) = northeastern, blue (24–32) = central, pink (33–34) = eastern, aqua (35–38) = western and red (39–41) = southern. The numbers in the circles are locality codes, as in Table S1. (**b**) The malaise trap in a typical forest setting.

This study used materials from the project Thailand Inventory Group for Entomology Research (TIGER project, 2006–2009). Collection permit no. 0002.3/5075 was issued for this study by the National Research Council. Insect specimens were collected during 2006–2009 from 41 collecting sites (Table S1) in 24 national parks, covering all regions in Thailand, which included 12, 10, 11, 2, 4 and 3 sites in the north, central, northeast, east, west, and south of the country, respectively (Figure 1a).

### 2.2. Collection Methods

Specimens of the longhorn beetle were collected by a standard Townes style malaise trap (Figure 1b) (width 100 cm, length 170 cm, and height 150 cm). A single trap in each collection site was operated for 12 consecutive months with a monthly service. Seasonal classification was based on rate of rainfall and air temperature by following the Thai Meteorological Department and dividing into three seasons; rainy (May–October), dry cold (November–February), and hot (March–April) for the northern, central, northeastern, eastern, and western regions of the country, while the southern region accounted for two seasons; hot (November–April) and rainy (May–October) [23]. Longhorn beetles were separated from other insect specimens at the laboratory in the Entomology Section of Queen Sirikit Botanic Garden (QSBGE), Chiang Mai, Thailand. 

### 2.3. Species Identification

Specimens of the longhorn beetle were identified into species based on their morphology by using the identification keys of Cerambycidae in Thailand [22,24], and related published papers on Cerambycidae in Thailand and its neighboring countries [25,26,27,28,29,30,31,32]. Since specimens could not be identified at the species level, they were treated as morphospecies at the generic level. All specimens represented in this study were deposited in the Entomology Section of the QSBGE, Chiang Mai Province, Thailand.

### 2.4. Statistical Analyses

Observed species richness and relative abundance of longhorn beetles by monthly samples from each site were recorded. Relative abundance was calculated by the total number of species occurrence divided by the total number of collections and presented in percentages. Frequency of species occurrence (SO) was calculated by the number sites with positive species divided by the total number of collection sites (*n* = 41) [33]. The Shannon_H index, Simpson_1–D, Evenness_e^H/S and Dominance_D were used to measure the biodiversity of the longhorn beetles in each collection site, and Chao1 richness estimator was used to estimate the total number of species in each one. The species accumulation curve (individual-based rarefaction), based on the Shannon_H index and taxa was used for comparing biodiversity between collection sites and to assess sampling adequacy. The SHE analysis that calculated the log for species abundance (ln S), Shannon H index and log for evenness (ln E = H − ln S) in all of the samples, from the first and last sample, were applied to assess variations of the community and species composition. The general linear model (GLM) was used to test the relationship between species richness of the longhorn beetle and elevation gradient. The matrix plot of monthly variation in species richness and abundance of the longhorn beetles in each national park was based on abundance data, and visualized as a contour map. Longhorn beetle species were represented by ≥ 10 individuals that were used for ordination analyses and the canonical correspondence analysis (CCA), and the site/species matrix was used to analyze species distribution related specifically to collection sites and environmental variables (forest types, elevations, and geographic regions). Correspondence analysis (CA) was used to interpret the relationship between longhorn beetles and forest types. Similarity among sampling sites was based on species composition data. Cluster analyses of the paired group (UPGMA) was based on the Bray–Curtis similarity index, with 1000 bootstraps carried out. All statistical analyses were performed by PAST version 4.03 [34], with statistical significance set at *p* < 0.05

## 3. Results

A total of 1376 specimens comprising 199 morphotaxa in 36 tribes of 6 subfamilies of Cerambycidae were identified from the 41 collection sites in 24 national parks over 6 geographic regions of Thailand (Table S2). Lamiinae was the subfamily with the highest number of 119 species, followed by Cerambycinae with 70, Dorcasominae with 3, Prioninae with 3, Lepturinae with 3 and Disteniinae with 1. Of these, five species with high relative abundance were *Nu. lenita*, *Pt.* sp1., *Ob.* sp.3, *Ac. pseudospeciosa* and *Ac. rustricatrix*, which represented 4.80%, 4.80%, 4.80%, 4.5%, and 4.43% of species, respectively. Six species had the widest distribution range, which represented a high percentage of species occurrence (% SO), with those having more than 30% being *Pt.* sp.1 (63.4%), *Mo. suffusa* and *Ac. rustricatrix* (39%), and *Niphona rondoni*, *Nu. Fricator,* and *Nu. lenita* (34.1%). The remaining 189 species had a % SO of less than 30%.

**Figure 2:**
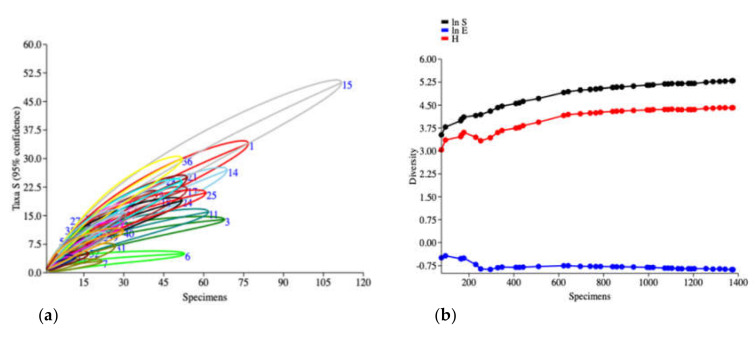
Biodiversity indices of longhorn beetles collected by the malaise trap from 41 collection sites in 6 geographic regions of Thailand. (**a**) Species cumulative curve (individually-based rarefaction) with 95% confidence interval. (**b**) SHE profiles represented the cumulative curve of ln S, H and ln E (*Y*-axis = diversity values of the log abundance, Shannon_H index and log evenness; (*X*-axis = number of specimens).

The species cumulative curve (individual-based rarefaction) was based on the number of species data (Figure 2a) showed non-asymptotic curve in each collection site referred to sampling insufficiency in all of the collection sites. Cumulative SHE profiles (Figure 2b) indicated that all of the indices were likely to curve asymptotically with a short period of initial variation, but all indices represented by high value, non-stable curve of SHE and more heterogeneity (represented by dots on the line) between collection sites (biotope) indicated that many more species of longhorn beetle remained undiscovered.

**Figure 3:**
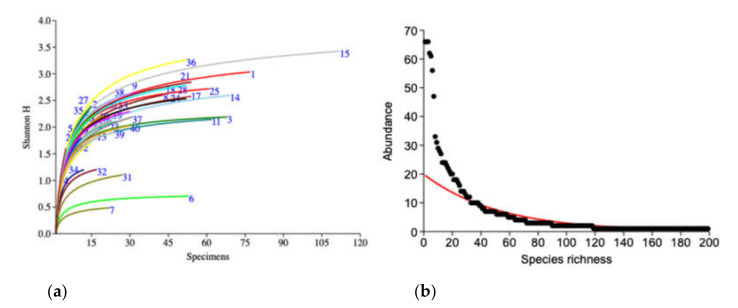
(**a**) Individual-based rarefaction curves (Shannon_H index) of longhorn beetles from 41 collection sites (number of curves that refers to the code of the collection sites is presented in Table S1). (**b**) Species abundance and distribution of longhorn beetle communities.

The biodiversity comparison between the collection sites (Figure 3a) and accumulation curve of the diversity index (Shannon_H index) revealed good proportion within all the collection sites between abundance among species by the asymptotic curve. Of these sites, no. 15 (Loei Forest Unit) had the highest diversity, followed by no. 36 (Panernthung) and no. 1 (Mae Fang Hotspring), while the lowest diversity was in site no. 7 (Kew Maepan).

Dominance was located in middle elevation (273–950 m asl) in 3 of the 41 collection sites: Loei Forest Unit, Mae Fang Hotspring and Panernthung, with all of them showing high species richness and diversity (Shannon_H index) at 50/3.42, 34/3.04 and 30/3.26 species and Shannon_H index, respectively (Table S3). These three dominant sites found 45% (90 species) of the total species. Of these, 7 species (7.8%) occurred at all of the sites and 8 (8.9%) were found in at least two, while of the remaining 75 species (83.3%) were specific to the collection sites.

A total of 199 morphospecies from 1376 specimens were captured in this study. Of these, 9 species were the most abundant; represented by 30–66 specimens, 29 were represented by 10–29 specimens and 51 by 3 or 4 specimens. In contrast, 81 species (40.7%) were represented as singletons (single individual) and 29 (14.5%) as doubletons (only two individuals collected) (Figure 3b). Observed species richness in each collection site ranged from 3 to 50 species. The estimated species richness (Chao1 estimator), which represented the number of species, was 6 and 1275 species at Vachiratharn water fall and Loei Forest Unit, respectively (Table S2).

**Figure 4:**
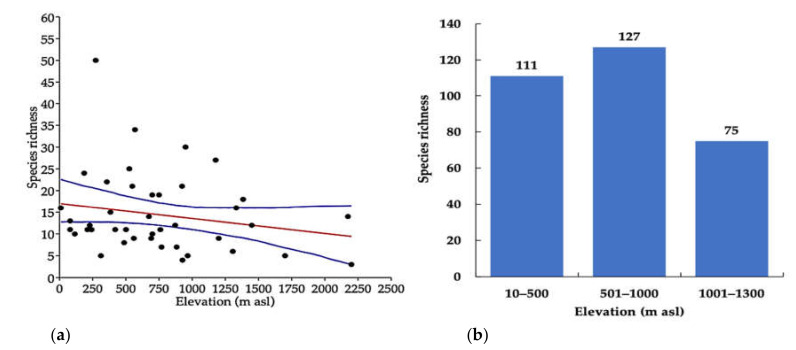
(**a**) Relationship of species richness and elevation. (**b**) Species richness along the elevation, (10–500 m asl = low elevation, 501–1000 m asl = middle elevation, 1001–1300 m asl = high elevation).

Relationship between species richness of the longhorn beetle and elevation gradient, the GLM showed a significantly negative relationship (y = −0.0034399x + 17) *p* < 0.05, R^2^ = 0.04) between species richness and elevation gradient (Figure 4a). Hump-shaped patterns by the number of species increasing at an elevation in the initial phase, peaked at middle elevation (501–1000 m asl) and then decreased at high elevation (1001–1300 m asl) (Figure 4b).

**Figure 5:**
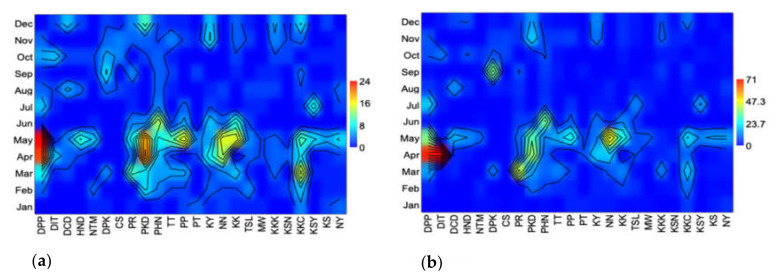
(**a**) Seasonal species richness. (**b**) Seasonal abundance of the longhorn beetle, collected by the malaise trap from 24 national parks in Thailand. (Abbreviations of national parks: DPP; Doi Phahompok, DIT; Doi Inthanon, DCD; Doi Chiang Dao, HND; Huai Nam Dang, NTM; Namtok Mae Surin, DPK; Doi Phu Kha, CS; Chae Son, PR; Phu Ruea, PKD; Phu Kradueng, PHN; Pa Hin Ngam, TT; Tat Tone, PP; Phu Phan, PT; Pha Taem, KY; Khao Yai, NN; Nam Nao, KK; Khao Kho, TSL; Thung Salaeng Luang, MW; Mae Wong, KKK; Khao Khitchakut, KSN; Khuean Srinagarindra, KKC; Kaeng Krachan, KSY; Khao Sam Roi Yot, KS; Khao Sok, and NY; Namtok Yong).

The seasonal species richness (Figure 5a) and abundance of longhorn beetles (Figure 5b) peaked in the northern, northeastern, central, eastern, and western regions of Thailand during the hot season (March–April) and continued into the early rainy season (May). Then they peaked again during the cold season (November–December), while species richness and abundance in the southern region were higher in the rainy season than the hot one.

**Figure 6:**
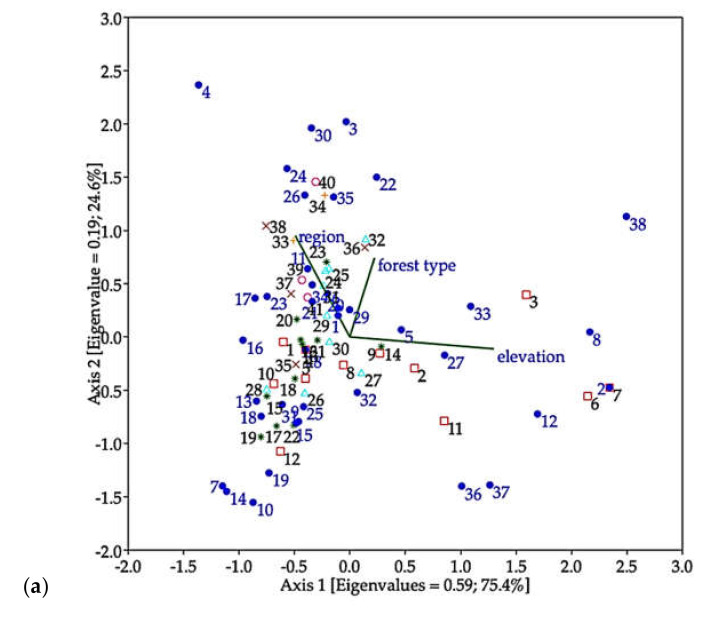

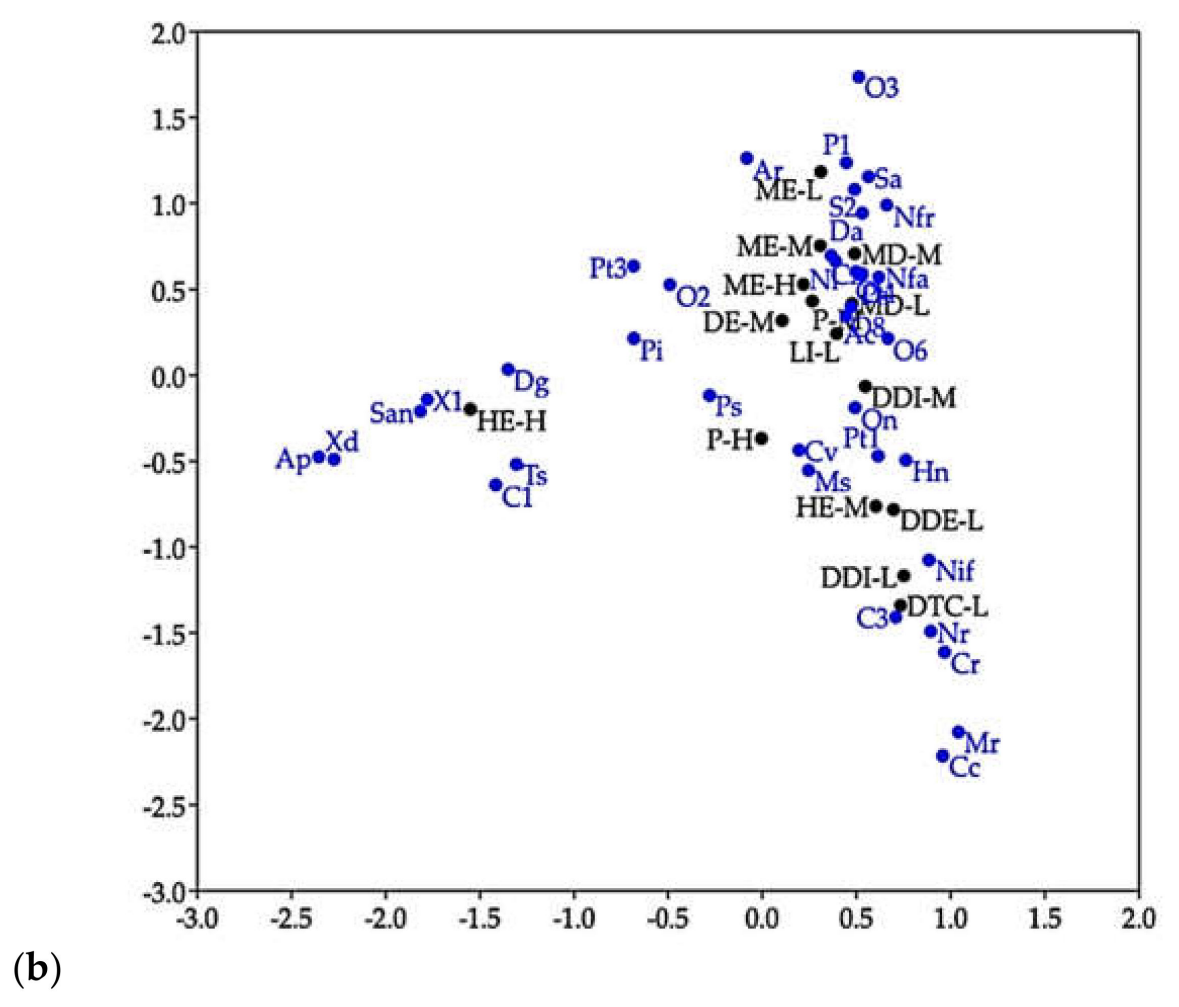
Ordination analyses. (**a**) Canonical correspondence analysis (CCA) representing the correlation between distribution of the longhorn beetle species (species 1 to 39) (species with more than 10 individuals) in collecting sites (sites 1 to 41), type of forest and elevation. (**b**) CA presenting the correlation between species of longhorn beetles and forest types located at different elevations.

In ordination analyses, CCA (Figure 6a) indicated that most species of longhorn beetles were distributed in regions and forest types, and it revealed that most of the species strongly correlated with forest located in low to middle elevation, while a group of seven species (Dg, Ts, C1, X1, San, Xd, and Ap) related to hilly evergreen forest located at high elevation (HE_H), as shown in Figure 6b.

**Figure 7:**
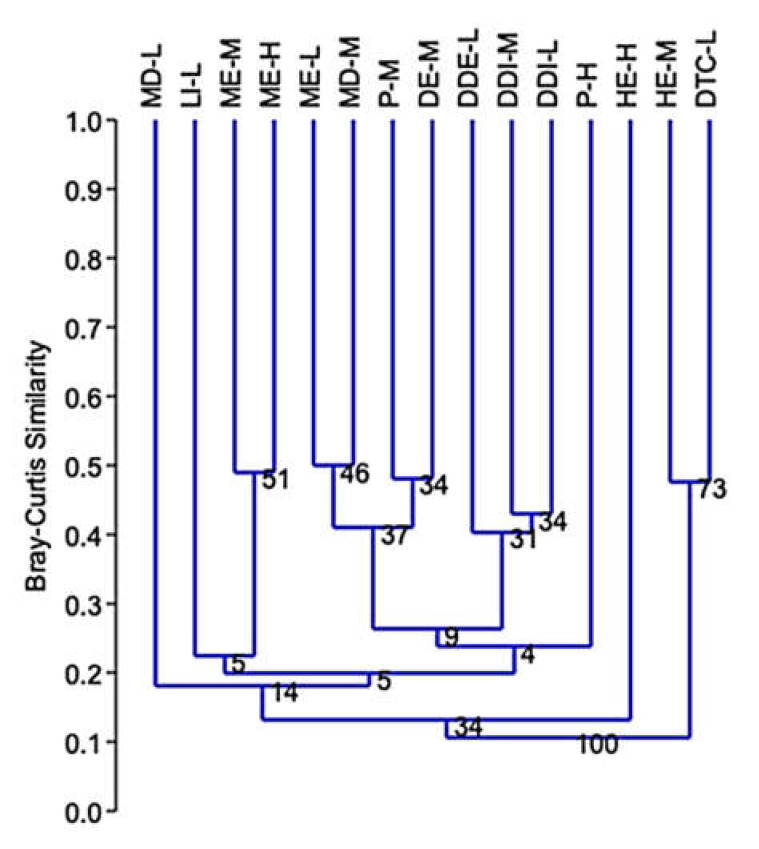
Similarity index using Bray–Curtis similarity (Cophen. Corr. = 0.78) based on species composition between forest communities. ME–H = Moist evergreen, high elevation; ME–M = Moist evergreen, middle elevation; ME–L = Moist evergreen, low elevation; HE–H = Hill evergreen, high elevation; HE–M = Hill evergreen, middle elevation; P–H = Pine, high elevation; P–M = Pine, middle elevation; DE–M = Dry evergreen, middle elevation; DDE–L = Dry deciduous, low elevation; MD–M = Mixed deciduous, middle elevation; MD–L = Mixed deciduous, low elevation; DDI–M = Dry dipterocarp, middle elevation; DDI–L = Dry dipterocarp, low elevation; DTC–L = Dipterocarpus, low elevation; LI–L = Limestone, low elevation.

Cluster analyses of Bray–Curtis similarity, based on species composition and abundance of longhorn beetle species, showed similar percentages between types of forest communities: ME–L and MD–M = 50%; ME–H and ME–M = 49%; ME–L and DE–M = 44%; and similarities between P–M and DE–M, and MD–M = 48% and 43%, respectively; HE–M and DDI–M = 47%; within the group of DE–M and MD–M, DDI–M, and DDE–L = 47%, 47%, and 41%, respectively; DDE–L and DDI–M, and DDI–L = 40% and 40%, respectively, and DDI–M and DDI–L = 43%, while MD–L, P–H, and HE–H had very low percentage similarities compared to other communities (Figure 7). 

## 4. Discussion

This study provides some data on the diversity, distribution and seasonal variation of longhorn beetles in Thailand with 199 morphotaxa being found using only malaise traps run over a period of 12 months from widespread locations in Thailand. This compares to other studies of cerambycids by using many collection methods in Thailand finding 87 [20], 62 [21], and 308 [22] species, respectively.

The results of this study revealed that a small proportion of actual species was found (15% or 199 species) of estimated (1275 species), suggesting that many of the 85% of longhorn beetle species in Thailand were not collected with support from the non-asymptotic species accumulation curve, based on Taxa S, as depicted in Figure 2a. This tendency was similar in previous studies that reported 20–50% of the estimated number of longhorn species not being recorded [13,35,36], and other insect studies showed various percentages of undiscovered species; for example, 24–44% of ant species in the western Amazonian rainforest of Ecuador [37]; 10–20% of Auchenorrhyncha and Diptera in the tropical forests of Thailand [38]; and 18% of the Empidoidea community in Doi Inthanon National Park, Thailand [39]. Therefore, additional intensive survey with several combined sampling methods are necessary in order to evaluate Cerambycidae fauna in Thailand. The estimated species richness of longhorn beetle from this study had similar trends to results reported from tropical countries, which have a high biodiversity of insects [40,41] such as Laos with 1156 species [42], Borneo, Indonesia with over 1300 species catalogued and more than 2000 expected [43], southern Yunnan, China, with 220 species [16], India with 1536 species [44], Australia with 536 Lamiinae species [2], and Mexico with more than 1600 species [14]. 

This study found that many species were captured in small numbers that represented a high percentage of singletons (40.7%) and doubletons (14.5%) (Figure 3b). Likewise, in other studies on communities of longhorn beetles, between 19.5 and 56% were presented by singleton species [11,35,45,46,47,48]. A study from southern Yunnan, China, indicated that 166 of 193 species from a forest area were represented by only 1–6 specimens [16]. Moreover, this trend is also found in other insect groups that had a high proportion of singletons in samples, especially tropical insect species due to insufficient sampling [41]. The small number of longhorn beetles caught during this study may be explained by the limitation of the collecting method used. Malaise traps are unbiased and passive, and do not use attractants or bait.

The collection sites in this study were located at various elevations that ranged between 10 and 2200 m, and classified into three zones, lowland (10–500 m asl), mid-elevation (501–1000 m asl), and highland (1001–1300 m asl). The results of this study indicated that the relationship between species richness and elevation was significantly negative with hump-shaped patterns and the number of species increasing by elevation at the initial phase; then peaking at the middle-elevation before decreasing at the high elevation. These characteristics are accepted as a general pattern [49] that is found in several examples of insect distribution, including that of plant species richness, which decreases with increasing elevation [36,50,51,52,53,54,55,56]. Middle elevations have many factors which make them suitable for insects such as optimal temperatures, greater host plant diversity or food availability. Furthermore, herbivorous beetles are a dominant group at this elevation in that they synchronize with host plants and correlate to precipitation, and the longhorn beetle has support of a biological niche [50,51,53,57].

The dominant collection sites with the greatest species richness and diversity index of the longhorn beetle (45% or 90 species of the total species from three localities) were Loei Forest Unit, Mae Fang Hotspring and Panernthung, which are located in lowland and mid-elevation (273–950 m asl). The first two sites were buffer zones located at the boundary of orchard areas and the third one was in natural forest. Both orchard areas or cultivated land and natural forest support high biodiversity of the longhorn beetle [16], and the effect of forest edges [58] on the distribution of saproxylic beetles was reported similarly, with higher species richness in temperate lowland than in montane forests [59]. In general, it can be assumed that cerambycid feeding on living, dying or dead wood as well as flowers is because these food supplies are more available in cultivated areas, which are associated with the cultivated activity of their local inhabitants. Moreover, both dominant sites of Loei Forest Unit and Mae Fang Hotspring were affected by wildfires that resulted in increase of food sources, i.e., tree decomposition [60].

The species richness and abundance of longhorn beetles peaked during the hot season (March–April) and early wet season (May). These seasonal patterns being similar to those reported by [22] from a study on cerambycids in northern Thailand. Likewise, a study report from a tropical dry forest in Mexico indicated that the greatest species richness and abundance of cerambycids were found during the rainy season [13], while a study from Illinois, USA, reported that the flight seasons of cerambycids start in mid-March, with abundance and species richness increasing to a peak in mid-July, and declining through to the end of August [47]. Moreover, phenology of the herbivorous beetle, Galerucinae, reaches the highest richness and abundance in tropical rain forest during the rainy season [53]. The seasonality of tropical insect abundance is influenced by the seasonal changes between dry and rainy seasons, which affects food source availability [13,53,57,61,62]. Food increases often coincide with leaf flush and flowering in the forest, which occur in the late dry to rainy season in Thailand.

When the community structure of Cerambycidae from this study was considered, the subfamily level was compared with previous work from northern Thailand [22], the proportionate number of species in each subfamily had a similar trend, with the greatest number of species were in the subfamily Lamiinae, followed by Cerambycinae and Prioninae, respectively. However, in a different number of subfamilies, three species in the unique subfamily, Dorcasominae, were found, while it previously showed that the subfamily Philinae and Aseminae had no positive result in these studies. Regarding different collecting methods, only the malaise trap was used in this study, but previous studies combined collecting techniques such as sweeping, light trap and direct searching in habitats appropriate for Cerambycidae.

Ordination analyses indicated that most species of longhorn beetles are distributed among specific regions and most of them strongly correlate with forest type located in low to middle elevation (there being little similarity between sampled communities). We conclude that cerambycids are specific to habitats with narrow distribution. However, equally, or more likely, is the possibility that these results are a consequence of the initial TIGER sampling plan being designed to specifically select dissimilar sites. Herein, the biodiversity data of cerambycids in Thailand came from malaise trap collection, which is passive, non-baited and acts as a flight barrier with no bias. The trap provided interesting data on not only the number of species and their abundance, but also their distribution data, which can be useful for agriculture and forestry including the challenge of conservation. The malaise trap is easy to use and set up and collect specimens. It is an effective trap for catching a large number and a wide variety of flying insects and is popularly used for monitoring insect diversity [38,39,63], parasitoid wasp (Hymenoptera), bee communities [64,65] and suitable for flying beetles [66]. Although malaise trap was not used as the best tool for collecting longhorn beetle specimens, it was used in conjunction with other methods for studying the diversity of Cerambycidae, which provide complete information, for example, light traps, direct searching on trees, beating, bowl traps, and sweeping, as reported by some previous studies [11,13,16,35,67]. For further study, this work recommends that the malaise trap should be combined with other methods which can help in gaining a better understanding and complete information of longhorn beetle fauna. It is reasonable that study on taxonomic revision should include the DNA technique in order to clarify and confirmed species status as a necessary topic in the future because many species are adopted in morphospecies. This study collected cerambycids from forested areas where cerambycid larvae likely play an important role as initial decomposers of trees. We hope to conduct further studies on collections from orchard, rubber or economic tree plantation in order to evaluate their effects on economic plant and host plant relationships. Finally, in this study, a huge number of insects were caught by a malaise trap. We sorted only the target insects (longhorn beetles). The remaining non-target insects including three predominant insects (represented about 95% of total specimens) belong to the order Diptera, Hymenoptera, Coleoptera, and other minor orders are also collected same as other previous reports [66,68,69,70,71,72]. We classified these non-target insects into the order or family and kept them in the good condition under low temperature at the Entomology section, Queen Sirikit Botanic Garden, where open and welcome for all entomologists who interests in using the material for study.

## 5. Conclusions

Quantitative data on the biodiversity and spatiotemporal variation of Cerambycidae in Thailand has been presented with this being the first report on the aspect of ecology. This study reports on the collection and examination of 1376 cerambycids collected from malaise traps from 41 sites over much of Thailand. We found a total of 199 morphospecies.

## Data Availability

The data presented in this study are available in supplementary materials.

## Supplementary Materials

The following are available online at https://www.mdpi.com/2075-4450/12/1/45/s1, Table S1: Collection sites of longhorn beetles in Thailand, Table S2: Species list and distribution of longhorn beetles, Table S3: Biodiversity data.
