# Supplementary material for: Biodiversity and Spatiotemporal Variation of Longhorn Beetles (Coleoptera: Cerambycidae) in Tropical Forest of Thailand
Source: Insects. 2021 Jan 8;12(1):45. doi: 10.3390/insects12010045 (PMC7827077; doi:10.3390/insects12010045)
Supplement: Supplementary file 1 [file insects-12-00045-s001.zip › Supplementary files/Supplementary_ Table S1_Collection sites of longhorn beetles in Thailand.docx]

**Supplementary 1**

**Table S1: Collection sites of longhorn beetles in Thailand.**

| **National Parks** | **Locality Name** | **Locality Code** | **Regions** | **Geographic Coordinate** | **Elevation m (msl)** | **Forest Type** |
| --- | --- | --- | --- | --- | --- | --- |
| Doi Phahompok | Mae Fang Hotspring | 1 | North | 19°57.961'N 99°9.355'E | 569 | Mixed Deciduous |
|  | Doi Phaluang | 2 | North | 20°1.06'N 99°9.581'E | 1449 | Hill Evergreen |
|  | Kiewlom | 3 | North | 20°3.549'N 99°8.552'E | 2174 | Hill Evergreen |
| Doi Inthanon | Vachiratharn water fall | 4 | North | 18°32.311'N 98°36.048'E | 700 | Dry Evergreen |
|  | Campground | 5 | North | 18°32.657'N 98°31.482'E | 1200 | Hill Evergreen |
|  | Check Point2 | 6 | North | 18°31.559'N 98°29.941'E | 1700 | Hill Evergreen |
|  | Kiew Mae Pan | 7 | North | 18°33.162'N 98°28.81'E | 2200 | Hill Evergreen |
| Doi Chiang Dao | Headquarter | 8 | North | 19°24.278'N 98°55.311'E | 549 | Dry Evergreen |
| Huai Nam Dang | Headquarter | 9 | North | 19°17.56'N 98°36.029'E | 1384 | Pine |
| Namtok Mae Surin | Headquarter | 10 | North | 19°21.593'N 97°59.254'E | 228 | Dry Deciduous |
| Doi Phu Kha | Headquarter | 11 | North | 19°12.326'N 101°4.765'E | 1332 | Hill Evergreen |
| Chae Son | Waterfall | 12 | North | 18°49.894'N 99°28.18'E | 487 | Mixed Deciduous |
| Phu Ruea | Checkpoint | 13 | Northeast | 17°27.829'N 101°21.36'E | 691 | Dry Dipterocarp |
|  | Huay Sai | 14 | Northeast | 17°29.92'N 101°20.51'E | 1177 | Pine |
| Phu Kradueng | Loei Forest Unit | 15 | Northeast | 16°56.65'N 101°48.9'E | 273 | Dry Deciduous |
|  | Forest Protection Unit5 | 16 | Northeast | 16°50.66'N 101°41.5'E | 420 | Dry Deciduous |
| Pa Hin Ngam | Headquarter | 17 | Northeast | 15°39.966'N 101°27.198'E | 357 | Dry Dipterocarp |
|  | Thung Dokkra Jeow | 18 | Northeast | 15°38.391'N 101°23.609'E | 750 | Dry Dipterocarp |
| Tat Tone | Tat Fah Waterfall | 19 | Northeast | 15°56.461'N 102°5.955'E | 242 | Dry Dipterocarp |
|  | Sab Somboon Forest Unit | 20 | Northeast | 16°1.059'N 101°58.603'E | 674 | Dry Dipterocarp |
| Phu Phan | Headquarter | 21 | Northeast | 16°48.618'N 103°53.476'E | 526 | Dry Evergreen |
| Pha Taem | Thung Luang | 22 | Northeast | 15°39.989'N 105°30.468'E | 238 | Dipterocarpus |
| Khao Yai | Dong Suer Paan | 23 | Northeast | 14°27.511'N 101°22.408'E | 760 | Moist Evergreen |
|  | Hnong Pakchee | 24 | Central | 14°27.119'N 101°21.482'E | 699 | Moist Evergreen |
| Nam Nao | Checkpoint | 25 | Central | 16°43.687'N 101°33.754'E | 924 | Pine |
|  | Hill Evergreen Forest | 26 | Central | 16°44.402'N 101°34.56'E | 883 | Hill Evergreen |
|  | Phu Kum Khao | 27 | Central | 16°42.47'N 101°35.26'E | 872 | Pine |
| Khao Kho | Khla Stream | 28 | Central | 16°39.257'N 101°7.945'E | 186 | Dry Deciduous |
|  | Mixed Deciduous Forest | 29 | Central | 16°32.546'N101°2.501'E | 560 | Mixed Deciduous |
| Thung Salaeng Luang | Gang Sopan Waterfall | 30 | Central | 16°52.46'N 100°49.67'E | 501 | Mixed Deciduous |
|  | Gang Wang Nam Yen | 31 | Central | 16°35.789'N 100°52.286'E | 769 | Pine |
| Mae Wong | Chong Yen | 32 | Central | 16°5.212'N 99°6.576'E | 1306 | Hill Evergreen |
| Khao Khitchakut | Prabaht Unit | 33 | East | 12°48.79'N 102°9.28'E | 80 | Moist Evergreen |
|  | Khao Prabaht Peak | 34 | East | 12°50.4'N 102°9.85'E | 927 | Moist Evergreen |
| Khuean Srinagarindra | Huay Mae Kamint | 35 | West | 14°38.312'N 98°59.643'E | 210 | Mixed Deciduous |
| Kaeng Krachan | Panernthung | 36 | West | 12°49.302'N 99°22.263'E | 950 | Dry Evergreen |
|  | Pa La-u Waterfall | 37 | West | 12°32.154'N 99°28.098'E | 384 | Moist Evergreen |
| Khao Sam Roi Yot | Tham Phraya Nakorn | 38 | West | 12°11.805'N 100°1.051'E | 10 | Limestone |
| Khao Sok | Headquarter | 39 | South | 8°54.896'N 98°31.81'E | 115 | Moist Evergreen |
| Namtok Yong | Campground Lavatory | 40 | South | 8°10.434'N 99°44.508'E | 80 | Moist Evergreen |
|  | TV Aerial | 41 | South | 8°14.262'N 99°48.289'E | 966 | Moist Evergreen |
